# Prevalence, incidence, and outcome of tuberculosis among young hospitalised children with acute illness in Sub-Saharan Africa and South East Asia

**DOI:** 10.7189/jogh.15.04338

**Published:** 2025-12-29

**Authors:** Mohammod Jobayer Chisti, Ezekiel Mupere, Abu Sadat Mohammad Sayeem Bin Shahid, John Mukisa, Gazi Md Salahuddin Mamun, Christopher Lwanga, Shamsun Nahar Shaima, Michael Atuhairwe, Md Farhad Kabir, Peace Aber, Willy Ssengooba, Lubaba Shahrin, Sayera Banu, Stephen M Graham, Judd L Walson, James A Berkley, Tahmeed Ahmed, Christina L Lancioni

**Affiliations:** 1 International Centre for Diarrhoeal Disease Research, Bangladesh (icddr,b) Dhaka, Bangladesh; 2 Childhood Acute Illness and Nutrition Network, Nairobi, Kenya; 3 Department of Paediatrics and Child Health, Makerere University College of Health Sciences, Kampala, Uganda; 4 Makerere University, Lung Institute, Kampala, Uganda; 5 Uganda-Case Western Reserve University Research Collaboration, Kampala, Uganda; 6 Department of Medical Microbiology, Makerere University, Kampala, Uganda; 7 Department of Paediatrics and Murdoch Children's Research Institute, Royal Children's Hospital, University of Melbourne, Melbourne, Victoria, Australia; 8 The Burnet Institute, Melbourne, Victoria, Australia; 9 Departments of International Health, Medicine and Pediatrics, Johns Hopkins University, Baltimore, USA; 10 Department of Global Health, University of Washington, Seattle, USA; 11 Centre for Tropical Medicine and Global Health, Nuffield Department of Medicine, University of Oxford, UK; 12 Clinical Research Department, KEMRI-Wellcome Trust Research Programme, Kilifi, Kenya; 13 Department of Pediatrics, Oregon Health and Science University, Portland, USA

## Abstract

**Background:**

Tuberculosis (TB) is a leading cause of paediatric morbidity and mortality. We sought to identify the prevalence of TB among children admitted to hospital with severe illness and to document incidence of TB, survival, and growth in the six months following discharge from hospital in two TB-endemic countries.

**Methods:**

We screened young children 2–23 months old enrolled in the Childhood Acute Illness and Nutrition Network cohort and admitted to hospitals in Bangladesh and Uganda for participation. Eligible children underwent comprehensive diagnostic evaluation for TB and were followed during hospitalisation and for six months post-discharge. We classified children as having bacteriologically confirmed, clinically diagnosed, or unlikely TB using standardised clinical definitions and microbiologic testing of sputum samples. We compared clinical and sociodemographic characteristics, and their associations with TB disease classification and six-month growth and survival.

**Results:**

Of 365 children eligible for participation, 17 (4.7%) were classified as bacteriologically confirmed, 46 (12%) clinically diagnosed, and 302 (83%) unlikely TB. Overall, 37 children were treated for TB; 18 (49%) during initial hospital admission and 19 (51%) during the six-month post-discharge period. All 17 children with bacteriologically confirmed TB survived through the post-discharge period and six-month survival did not differ by TB disease classification. Children with clinically diagnosed TB were more likely to be malnourished at enrolment, and anthropometric Z-scores were significantly lower among children classified as clinically diagnosed compared to unlikely TB throughout the post-discharge period.

**Conclusions:**

One in 10 children hospitalised in two distinct TB-endemic countries required treatment for TB, with half of these TB treatment courses initiated within a six-month observational period following hospital discharge. Children who meet criteria for clinically diagnosed TB are at increased risk of poor growth during the six months following hospitalisation, regardless of TB treatment initiation. These unique findings highlight the need for post-discharge monitoring for both TB and growth trajectories among recently hospitalised young children in TB-endemic settings.

Tuberculosis (TB) is a leading cause of morbidity and death in all ages globally. An estimated 1.3 million children (<15 years) developed TB in 2022, with 214 000 deaths reported. The majority (>80%) of paediatric TB deaths occur in young children <5 years [1]. *Mycobacterium tuberculosis* infection can cause severe TB disease in young children, particularly infants <2 years old [2]. Diagnosis in this age group is challenging due to the paucibacillary nature of paediatric TB, the non-specific and often acute disease presentation, and the difficulty in collecting respiratory samples for diagnostic testing [3]. Unfortunately, a missed diagnosis of TB in young children increases the risk of disseminated disease and death [4]. A recent modelling study suggested that up to 96% of children who died from TB never received treatment [5]. These findings suggest that TB must be considered among all seriously ill young children living in endemic settings, and that healthcare providers must maintain a high index of suspicion for TB following hospital discharge for children initially not diagnosed with TB who do not demonstrate robust clinical recovery and expected growth outcomes.

Malnutrition is commonly associated with TB in children; a systematic review reported that up to half of young children diagnosed with TB exhibit severe malnutrition [6]. This association is bidirectional as children with severe malnutrition are at high risk for TB following exposure, while TB may contribute to childhood malnutrition [7]. Malnourished children with TB may not present with overt signs and symptoms of disease, further adding to diagnostic challenges in young children. Malnutrition is a risk factor for mortality in children with TB [8], with deaths reported in 9–20% of malnourished children in some hospital settings [9,10]. Thus, even among young children treated for TB, there remains a risk of poor outcomes with advanced disease presentation. The impact of TB and TB treatment on long-term growth trajectories remains poorly understood, although a recent prospective long-term cohort study reported an association between TB, stunting and wasting [11]. Additional data on weight gain and linear growth among malnourished children treated for TB are limited despite the recognition that weight gain is an important marker of a successful treatment response.

We hypothesised that TB is commonly under-recognised during hospitalisation among young children in TB-endemic settings, with the emergence of signs and symptoms of disease during the post-discharge period. We also hypothesised that bacteriologically confirmed and clinically diagnosed TB would be associated with mortality and poor growth outcomes. Using a six-month observational study conducted in two TB-endemic settings, we aimed to identify the prevalence of TB during hospitalisation, determine the incidence of TB during the six-month post-discharge period, and evaluate the six-month post-discharge survival and growth outcomes among young children with bacteriologically confirmed, clinically diagnosed, and unlikely TB.

## METHODS

### Study design and settings

Young children were enrolled in the Childhood Acute Illness and Nutrition (CHAIN) Network cohort study, which systematically enrolled young children aged 2–23 months with acute illness at nine sites across sub-Saharan Africa and South Asia between 20 November 2016 and 31 January 2019. Children enrolled at the Dhaka Hospital of icddr,b, Bangladesh, and Mulago National Referral Hospital, Kampala, Uganda, were eligible. Children were enrolled at hospital admission, followed daily through hospital discharge, and underwent scheduled follow-up visits at days 45, 90, and 180 post-discharge. Both sites strictly adhered to the same structured case report forms and standard operating procedures for performing TB diagnostic evaluations throughout the study, including during post-discharge follow-up assessments. Detailed clinical and sociodemographic data were collected throughout the study, as were survival and growth since discharge. All CHAIN sites underwent extensive training and monitoring to ensure harmonisation of all study procedures and data collection; details of the CHAIN observational cohort protocol have been previously published [12].

### Study population

Young children being admitted to hospital with acute illness of all nutritional status were eligible for entry into the primary CHAIN cohort, with targeted enrolment in a 2:1:2 ratio based on: no wasting (mid upper arm circumference (MUAC)≥12.5 cm for age ≥6 months or MUAC≥12.0 cm for age <6 months); moderate wasting (MUAC 11.5–12.5 cm for age ≥6 months or MUAC 11.0–12.0 cm for age <6 months); and severe wasting (MUAC<11.5 cm for age ≥6 months or MUAC<11.0 cm for age <6 months) or bilateral pedal oedema [13].

**Figure 1:**
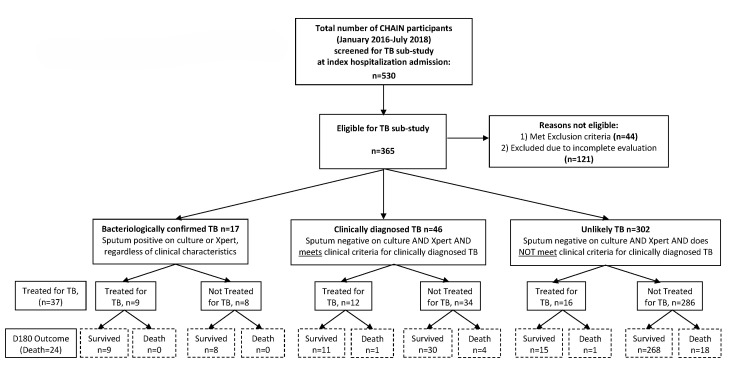
Study overview and outcome summary.

All CHAIN participants enrolled at the two TB sub-study sites underwent additional screening to determine eligibility for TB sub-study participation, including assessment for TB contacts, tuberculin skin test (TST) placement, and two-view chest x-rays (CXR). We excluded children who had received anti-TB therapy for >72 hours before enrolment, and children without wasting by the CHAIN MUAC criteria and all of the following: negative TST, normal two-view CXR, no known TB contact in past 12 months, human immunodeficiency virus (HIV)-uninfected, and a confirmed diagnosis of malaria or diarrhoea that improved within 72 hours of admission. Children who did not have their TB screening and eligibility completed within 72 hours of enrolment in the primary CHAIN study were excluded from this sub-study analysis due to an incomplete evaluation (**Figure 1**; Tables S1 and S2 in the **Online Supplementary Document**).

### Clinical management

Hospital clinicians determined clinical management and treatment decisions. All young children enrolled in the primary CHAIN observational study underwent routine clinical evaluation, testing, and treatment in accordance with local clinical guidelines and standard of care. Upon enrolment in the primary CHAIN cohort study at hospital admission, all children also underwent a comprehensive medical examination by trained study physicians, and laboratory assessments (complete blood cell count with differential, plasma glucose, rapid malaria test, serum electrolytes, renal and liver function tests, and HIV testing), with strict adherence to CHAIN standard operating procedures. As part of the primary CHAIN study, each child in our study population received an illness severity score based on data collected at the time of their index hospitalisation [13]. Trained study staff systematically collected comprehensive data on clinical diagnoses, treatment initiation, and socio-demographics, including food security, using structured case report forms.

### TB diagnostics

#### Sputum collection and laboratory investigations

Eligible children underwent one sputum induction for acid fast bacilli (AFB) smear and culture, and immediate molecular rapid diagnostic testing (Xpert MTB/RIF or Xpert Ultra). Trained research physicians collected a single induced sputum specimen following standardised operating procedures based on published protocols [14]. Sputum underwent standard processing [15] before AFB smear, culture and Xpert testing following routine protocols [16,17]. They used traditional techniques to conduct both liquid (BACTEC MGIT) and solid Lowenstein-Jensen slant AFB cultures, and to perform first-line antibiotic susceptibility testing of isolated *M. tuberculosis* strains [16].

We analysed induced sputum using Xpert MTB/RIF or Xpert Ultra (as available) according to the manufacturer's instructions. For purposes of classification and analysis, ‘Xpert’ test includes either Xpert MTB/RIF or Xpert Ultra. Sputum Xpert MTB/RIF testing was initially performed as the Xpert Ultra assay was not available at study onset. In brief, the specimen was mixed with reagent buffer in a 2:1 ratio. The sealed container containing the mixture was manually shaken twice during a 15-minute incubation at room temperature, then 2 mL of the inactivated mixture was transferred to the test cartridge. The cartridge was placed into the test platform, and the result was automatically generated and recorded in approximately 2 hours [16,17]. Induced sputum samples were analysed immediately following collection by either Xpert MTB/RIF or Ultra, once the Xpert Ultra assay became available in December 2017. For 34 children, residual sputum samples initially tested on the Xpert MTB/RIF platform were cryopreserved and retested using Xpert Ultra once available.

#### TST

Trained research physicians performed TST by injecting five tuberculin units/0.1 ml of PPD-S intra-dermally on the volar aspect of the left forearm, almost parallel to the skin surface. After 48–72 hours, the site was assessed for induration and considered positive if induration was ≥10 mm in diameter; for children with severe wasting or HIV-infection, induration of ≥5 mm was considered positive [18].

#### CXR

Children underwent anteroposterior and lateral CXRs. Using a standardised CXR evaluation form (Annexe S1 in the **Online Supplementary Document**), both a radiologist and study physician independently interpreted CXRs as normal or abnormal and the specific abnormalities identified.

Findings considered consistent with TB were [19]: parenchymal disease (*i.e.* air space opacification or consolidation, air bronchogram, infiltrates, lobar collapse), pleural effusion, miliary pattern and/or hilar or mediastinal lymphadenopathy (*i.e.* soft tissue density with or without airway compression) were reported as abnormal. Any concordant interpretation (*i.e.* normal or abnormal) was taken as the final CXR result. In cases of discordant interpretation, a third physician provided additional evaluation, and the interpretation agreed with either of the initial readers was taken as the final result.

#### TB treatment and follow-up

Initiation of treatment for TB disease during hospitalisation was based on national and international guidelines, per the discretion of hospital clinicians and not dictated by research protocols. Treating physicians received laboratory test reports generated as part of the CHAIN TB sub-study as soon as they were available. At both participating hospitals, clinicians utilised World Health Organization (WHO) diagnostic and treatment guidelines for TB in children [20] available during the study period, as well as guidelines for clinical management of severe malnutrition [21]. Research clinicians followed children discharged alive for six months after hospital discharge and systematically screened them on post-discharge days 45, 90, and 180 for TB signs, symptoms, and exposures. If any child not initially diagnosed with TB developed suggestive signs and symptoms during scheduled post-discharge follow-up assessments or unscheduled sick visits/hospital re-admissions, the TB diagnostic evaluation was repeated. Referrals for initiation of TB treatment, and/or hospital re-admission, were made based on the results of investigations. Loss-to-follow-up rates at both recruitment sites were <2%.

#### TB classification

Following completion of the six-month observational period, research personnel performed TB classification retrospectively using standardised criteria, regardless of clinical diagnosis and/or TB treatment initiation. The criteria applied for research classification of paediatric TB were the Bangladesh National TB Programme Guidelines [18], adapted from WHO guidelines [20]. We defined bacteriologically confirmed TB as MTB isolated from a respiratory sample by either AFB microscopy, culture, or Xpert assay, regardless of clinical presentation. We defined clinically diagnosed TB as negative sputum testing (AFB microscopy, culture, and Xpert) with at least three clinical signs and symptoms of TB18 including: cough and/or fever for ≥2 weeks, severe wasting or recent weight loss, reduced activity and playfulness, persistent lymphadenopathy, known TB contact within the past 12 months, positive TST, and/or CXR suggestive of TB. Lastly, we defined unlikely TB as negative sputum testing (AFB microscopy, culture, and Xpert) and less than three clinical signs and symptoms of TB. Initiation of TB treatment and responses to TB treatment were not utilised for TB classification. The timing of TB diagnosis was distinguished as occurring during the index hospitalisation or the six-month post-discharge period.

### Statistical analysis

To describe the baseline characteristics of participants, including demographic information, clinical characteristics at admission, and laboratory findings, we used descriptive statistics such as mean, standard deviation, median, interquartile range, frequency, and percentage. To compare categorical variables between groups of bacteriologically confirmed, clinically diagnosed, and unlikely TB, we used the χ^2^ test or Fisher exact test, as appropriate. We used the *t* test or the Mann-Whitney test for comparing continuous variables. We used a one-way ANOVA or a Kruskal-Wallis test (with *post hoc* analysis) to assess the statistical significance of differences among the groups (bacteriologically confirmed, clinically diagnosed, and unlikely TB).

We performed longitudinal data analysis using generalised estimating equations (GEEs) to examine the association between anthropometric changes over time across the three participant groups. The analysis was adjusted for age, sex, household wealth, food insecurity and TB treatment status. All GEE analyses used a Gaussian distribution with an identity link function and an exchangeable correlation matrix for the estimation.

Anthropometric measurements collected longitudinally over 180 days post-discharge provided critical indicators of growth since discharge (Table S3 in the **Online Supplementary Document**). We employed GEE as our data set included repeated measures and was longitudinal in design. GEE is well-suited for handling correlated data, such as repeated measures from the same individuals, and allows for robust estimation of standard errors. This method enabled us to accurately assess growth from discharge onward while accounting for intra-subject correlations over the follow-up period.

To test potential risk factors associated with the TB classification cohort, we used multinomial logistic regression to analyse the association between TB status (bacteriologically confirmed, clinically diagnosed, and unlikely TB) and outcomes of interest. Multinomial logistic regression is particularly appropriate for the comparison of more than two categories, with the reference category defined as ‘unlikely TB’. This method allowed us to estimate odds ratios for outcomes – unlikely TB *vs.* bacteriologically confirmed TB and unlikely TB *vs.* clinically diagnosed TB – while adjusting for relevant covariates identified through bivariate analysis. This approach allowed a direct assessment of the burden of TB across different groups in an adjusted model.

Both GEE and multinomial logistic regression are robust to missing data, notably when data are missing at random, as is often the case in longitudinal studies. GEE adjusts for the correlation of repeated measures, and multinomial logistic regression can accommodate missing data via maximum likelihood estimation, assuming missingness is missing at random. Both statistical methods allowed adjustment for covariates based on our bivariate analysis. This helped ensure that our results were not confounded by known risk factors and other variables that might influence the outcomes.

All independent variables hypothesised *a priori* to be clinically important (age, sex, household wealth and food insecurity, nutritional status, readmission required, failure to thrive, illness severity score) were examined in univariate multinomial logistic regression and retained in the multivariable models regardless of statistical significance. We assessed multicollinearity among independent variables using variance inflation factors. A probability of less than 0.05 was considered statistically significant. The strength of association was determined by estimating the adjusted odds ratios and their 95% confidence intervals (CIs). During the analysis, we weighted the sample to account for selection bias introduced by the stratified enrolment for CHAIN inclusion. All statistical analyses were performed using STATA, version 15.0 (StataCorp, College Station, Texas, USA).

## RESULTS

### Participant recruitment

From January 2016 to July 2018, 530 children underwent screening for eligibility for the TB sub-study at the Dhaka and Kampala CHAIN study sites (**Figure 1**). We excluded 44 children who did not meet the inclusion criteria. Of the remaining eligible children, 122 had incomplete evaluations for TB disease and were excluded from analysis (Table S1 in the **Online Supplementary Document**). Children excluded due to incomplete evaluations were more likely to be malnourished, HIV-exposed, present with more severe illness at initial hospitalisation, and were more likely to have died during their index hospitalisation as compared to children with complete evaluations who were included in the analysis (Table S2 in the **Online Supplementary Document**). Most children with incomplete evaluations were enrolled at the Kampala CHAIN site. Factors associated with incomplete evaluation included: critically ill children who could not be transported for CXR or have sputum induction performed within 72 hours of enrolment, and children with less severe illness discharged from the hospital before completion of screening procedures (Table S1 in the **Online Supplementary Document**). Children included in the analysis set had more frequent readmissions than those excluded due to incomplete evaluation (Table S2 in the **Online Supplementary Document**).

### Final TB study classification and characteristics

All TB classifications were performed for analytic purposes only after completion of the primary CHAIN cohort study and were not utilised to guide clinical decision-making, including initiation of TB treatment. Of 365 children included in the TB sub-study, 17 (4.7%) were classified as having bacteriologically confirmed TB; 46 (13%) as having clinically diagnosed TB; and 302 (83%) as having unlikely TB (**Figure 1**). Five children with bacteriologically confirmed TB, five children with clinically diagnosed TB, and eight children classified as unlikely TB were diagnosed and treated for TB during their index hospitalisation, representing 49% of all children treated for TB.

Demographics and clinical characteristics of children at enrolment are shown in Table 1. Compared to children with clinically diagnosed TB, children with bacteriologically confirmed TB were less likely to be severely wasted, have prolonged cough (>14 days), and have an abnormal CXR [19] consistent with TB. Children with bacteriologically confirmed TB were also less likely to have an illness severity score classified as moderate when compared to those with clinically diagnosed disease. Compared to children with bacteriologically confirmed or unlikely TB, clinically diagnosed TB cases were more likely to present with typical TB-related signs and symptoms, including prolonged cough, have an abnormal CXR or be severely wasted. These were expected findings as they form the basis for classification as clinically diagnosed TB. HIV infection was uncommon overall (3% of the total cohort), but noted to be more prevalent among children with clinically diagnosed TB.

**Table 1:** Participant demographic and clinical characteristics by TB disease classification

	Total (n = 365)	Bacteriologically confirmed (n = 17)	Clinically diagnosed (n = 46)	Unlikely (n = 302)	*P*-value
**Female (%)**	145 (39.7)	7 (41.2)	17 (37.0)	121 (40.1)	0.915
**Age of the child in months**					
2–11	122 (33.4)	5 (29.4)	17 (37.0)	100 (33.1)	0.821
12–23	243 (66.6)	12 (70.6)	29 (63.0)	202 (66.9)	0.821
**Currently breastfeeding**	215 (58.9)	9 (52.9)	21 (45.7)	185 (61.3)	0.118
**Caregiver education**					
None	99 (27.2)	7 (41.2)	10 (22.2)	82 (27.2)	0.326
Primary	170 (46.7)	8 (47.1)	20 (44.4)	142 (47.0)	0.949
Above primary	95 (26.1)	2 (11.8)	15 (33.3)	78 (25.8)	0.221
**Nutritional status**					
No wasting	65 (17.8)	4 (23.5)	1 (2.2)	60 (19.9)	0.003†
Moderate wasting	81 (22.2)	5 (29.4)	7 (15.2)	69 (22.9)	0.392
SWK	219 (60.0)	8 (47.1)	38 (82.6)	173 (57.3)	0.003†
**TB contact in the past 12 months**	15 (4.1)	1 (5.9)	9 (20.0)	5 (1.7)	<0.001*
**BCG**	293 (80.3)	13 (76.5)	36 (78.3)	244 (80.8)	0.793
**Diarrhoea>14 days**	14 (3.8)	1 (5.9)	4 (8.7)	9 (3.0)	0.106
**Cough >14 days**	44 (12.1)	1 (5.9)	21 (45.7)	22 (7.3)	<0.001†
**Poor feeding/weight loss**	89 (24.4)	5 (29.4)	18 (39.1)	66 (21.9)	0.037*
**HIV status**					
HIV-exposed	19 (5.2)	0 (0)	5 (10.9)	14 (4.6)	0.158
HIV-infected	11 (3.0)	0 (0)	6 (13.0)	5 (1.7)	0.002*
**Abnormal CXR consistent with TB **	309 (84.9)	13 (81.3)	46 (100.0)	250 (82.8)	0.001†
**TST positive**	27 (10.0)	4 (23.5)	14 (56.0)	9 (3.9)	<0.001‡
**Died**	24 (6.6)	0	5 (10.9)	19 (6.3)	
Death during initial hospitalisation	15 (4.1)	-	4 (8.7)	11 (3.6)	0.245
Death within six months post-discharge	9 (2.5)	-	1 (2.2)	8 (2.7)	1.000
**Illness severity score**					
Low	223 (61.1)	10 (58.8)	16 (34.8)	197 (65.2)	0.001*
Medium	86 (23.6)	3 (17.7)	23 (50.0)	60 (19.9)	0.001†
High	56 (15.3)	4 (23.5)	7 (15.2)	45 (14.9)	0.540
**Household assets index**					
Least poor	4 (1.1)	1 (5.9)	0 (0)	3 (1.0)	0.246
Fourth	40 (11.0)	2 (11.8)	6 (13.0)	32 (10.6)	0.726
Middle	77 (21.1)	4 (23.5)	14 (30.4)	59 (19.5)	0.197
Second	121 (33.2)	6 (35.3)	14 (30.4)	101 (33.4)	0.905
Poorest	123 (33.7)	4 (23.5)	12 (26.1)	107 (35.4)	0.303
**Household food insecurity**					
Low	236 (64.7)	10 (58.8)	23 (50.0)	203 (67.2)	0.066
Medium	85 (23.3)	4 (23.5)	16 (34.8)	65 (21.5)	0.129
High	44 (12.1)	3 (17.7)	7 (15.2)	34 (11.3)	0.490

BCG – *Bacillus Calmette–Guérin*, CXR – chest x-rays, SWK at D180– presence of severe wasting and/or Kwashiorkor at day 180 follow-up, TB – tuberculosis, TST – tuberculin skin test

*Post hoc analysis was conducted for clinically diagnosed *vs.* unlikely.

†*Post hoc* analysis was conducted for bacteriologically confirmed *vs.* clinically diagnosed and clinically diagnosed *vs.* unlikely.

‡*Post hoc* analysis was conducted for bacteriologically confirmed *vs.* clinically diagnosed, bacteriologically confirmed *vs.* unlikely, and clinically diagnosed *vs.* unlikely (All groups are different).

### Identification of TB requiring treatment following hospital discharge

Children who were discharged alive following initial hospitalisation (n = 350) underwent standardised re-screening for emergence of signs and symptoms of TB and new TB exposures on post-discharge days 45, 90, and 180, as well as during any hospital re-admission or unscheduled sick visits.

Four children with bacteriologically confirmed TB, seven with clinically diagnosed TB, and eight classified as unlikely TB were diagnosed and treated for TB during the post-discharge period, representing 51% of all children treated for TB. One child with bacteriologically confirmed TB was identified during a hospital re-admission, and three during scheduled follow-up visits (24% of bacteriologically confirmed TB). Two children with clinically diagnosed TB were identified during hospital re-admission, and five during a 180-day follow-up visit (15% of clinically diagnosed TB). Eight (2.6%) children with unlikely TB received a diagnosis of clinical TB and were initiated on treatment during the post-discharge period (Table S4 in the **Online Supplementary Document**).

### Detection of MTB in sputum

Overall, 17 (4.7%) children had MTB detected in a sputum sample and were bacteriologically confirmed with TB: 4 by Xpert MTB/RIF, 12 by Xpert Ultra, and one by both (**Table 2**). Notably, there were no differences in clinical symptoms, demographics, or epidemiologic exposures among children with bacteriologically confirmed TB when compared to children with negative sputum test results. However, children with positive sputum testing were more likely to receive TB treatment. Most positive sputum assays were identified using the Xpert Ultra assay and were reported at a ‘trace’ level of detection (Table S5 in the **Online Supplementary Document**). Only one child had MTB detected in an induced sputum sample by AFB culture.

**Table 2:** Six-month outcomes among children with bacteriologically confirmed, clinically diagnosed, and unlikely TB*

	Total	Bacteriologically confirmed TB	Clinically diagnosed TB	Unlikely TB	*P*-value
**SWK at D180 (total)**	30 (9.3)	0 (0)	6 (16.2)	24 (8.9)	0.180
SWK at D180 treated for TB	5 (16.7)	0 (0)	2 (33.3)	3 (12.5)	0.254
**Re-admission**	66 (18.1)	5 (29.4)	10 (21.7)	51 (16.9)	0.336
Re-admissions treated for TB	8 (12.1)	3 (60.0)	1 (10.6)	4 (7.8)	0.021†
**Death (index admission or post-discharge)**	24 (6.6)	0 (0)	5 (10.9)	19 (6.3)	0.308
Treated for TB before death	2 (8.3)	0 (0)	1 (20.0)	1 (5.3)	0.380

SWK – severe wasting and/or Kwashiorkor, TB – tuberculosis

*All values are presented as numbers (percentages) unless specified otherwise.

†*Post hoc* analysis of bacteriologically confirmed *vs.* unlikely TB.

### Treatment initiation, survival, and growth outcomes among study participants

Of 17 children with bacteriologically confirmed TB, only five had their confirmatory test results available in real time to inform clinical decision-making. Nine of 17 children with confirmed disease (53%) were treated for TB. There were no hospital or post-discharge deaths among any child with bacteriologically confirmed TB, including the eight who were not treated for TB (**Table 3**). Children not treated with bacteriologically confirmed TB all had sputum reported ‘trace’ positive by Xpert Ultra using a cryopreserved sample not reported in real-time, minimal to no signs or symptoms of TB reported, and were well nourished at their 180-day post-discharge assessment (Table S6 in the **Online Supplementary Document**).

**Table 3:** Anthropometric changes up to day-180 post-discharge*

	MUAC		HAZ		WAZ		WHZ	
	**Coef (95% CI)**	***P*-value**	**Coef (95% CI)**	***P*-value**	**Coef (95% CI)**	***P*-value**	**Coef (95% CI)**	***P*-value**
**TB category**								
Unlikely	ref		ref		ref		ref	
Bacteriologically confirmed	0.11 (−0.24, 0.47)	0.538	−0.05 (−0.45, 0.36)	0.815	0.03 (−0.36, 0.42)	0.885	0.01 (−0.36, 0.38)	0.949
Clinically diagnosed	−1.00 (−1.26, −0.75)	<0.001	−0.80 (−1.09, −0.50)	<0.001	−0.95 (−1.23, −0.67)	<0.001	−0.74 (−1.01, −0.48)	<0.001
**TB treatment**								
Not treated	ref		ref		ref		ref	
Treated	−0.30 (−0.56, −0.05)	0.020	−0.11 (−0.39, 0.18)	0.474	−0.42 (−0.69, −0.14)	0.003	−0.49 (−0.75, −0.22)	<0.001

Coef – coefficient, CI – confidence interval, HAZ – height for age Z-score, MUAC – mid-upper arm circumference, WAZ – weight for age Z-score, WHZ – weight for height Z-score, ref – reference, TB – tuberculosis

*All results adjusted for age, sex, time, household food insecurity, and asset quintile.

There were no statistically significant differences in the outcomes of death (in-patient or post-discharge), hospital re-admission, or the presence of severe wasting 180 days post-hospital discharge among children classified as bacteriologically confirmed, clinically diagnosed or unlikely TB (**Table 3**). Of children classified with clinically diagnosed TB, 26% (n/N = 12/46) were treated for TB, one of whom died. Of 34 children classified with clinically diagnosed TB who were not treated for TB, four died while receiving antibiotics for non-TB pneumonia. The five deaths among children classified with clinically diagnosed TB were complicated by severe wasting. Of 302 children classified as unlikely to have TB, 16 (5.3%) received TB treatment and one died. Of 285 children classified as unlikely TB who were not treated, 18 died. (**Figure 1**).

**Figure 2:**
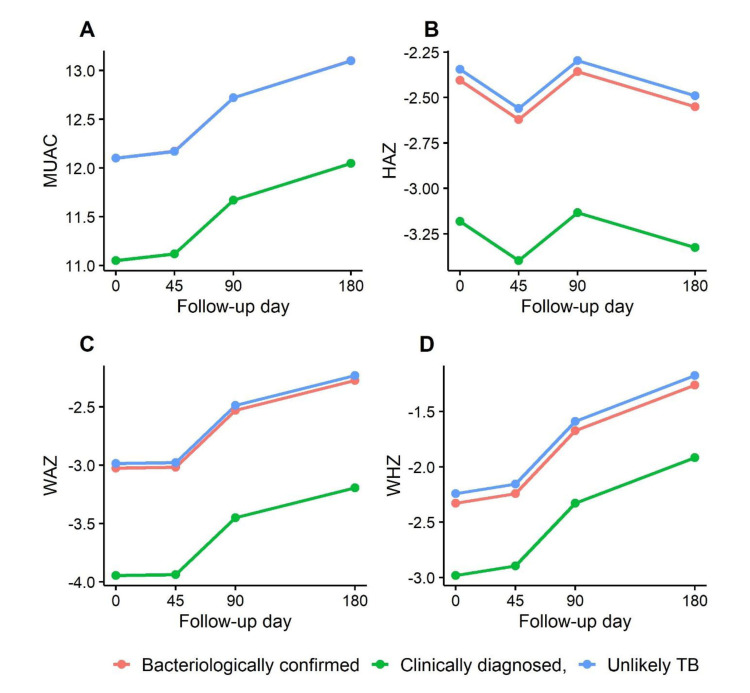
Six-month anthropometric changes among children classified as bacteriologically confirmed, clinically diagnosed, and unlikely TB.

The changes in MUAC (1.96 *vs.* 1.79, *P* = 0.728), WAZ (1.24 *vs.* 0.83, *P* = 0.392), WLZ (1.89 *vs.* 1.25, *P* = 0.225), and LAZ (−0.33 *vs.* −0.42, *P* = 0.799) between discharge and day 180 among children with bacteriologically confirmed TB who were and were not treated, were comparable with. Compared to children with bacteriologically confirmed TB or unlikely TB, children with clinically diagnosed TB had significantly lower MUAC at initial hospital admission and 180 days post-discharge. LAZ, WAZ, and WLZ scores at all follow-up time points were significantly lower among children with a clinically diagnosed TB diagnosis than among those with an unlikely TB diagnosis (**Figure 2**; Table S3 in the **Online Supplementary Document**).

Growth trajectories among children with clinically diagnosed TB were significantly reduced when compared to other groups after adjusting for age, sex, and time (**Table 4**). These differences remained significant after stratification based on receipt of TB treatment among all children. We also analysed anthropometric measures over time in children with different TB classifications who were severely wasted *vs.* not wasted (Table S3 in the **Online Supplementary Document**). Here, we compared WAZ, LAZ, and WHZ at each study time point, and compared weight gain and linear growth over time among children with and without severe wasting at hospital admission within each TB classification cohort. Similar to findings reported for the main CHAIN cohort [22], we observed robust catch-up weight gain among children with severe wasting during the early post-discharge period who were classified as unlikely TB. Notably, children with severe wasting and confirmed or clinically diagnosed TB did not demonstrate accelerated post-discharge weight gain when compared to those without severe wasting. When examining changes in age- and sex-appropriate international Z-scores for children, we observed that the mean WAZ in the clinically diagnosed TB cohort remained below the threshold for severe wasting (≤−3) at day 180 post-discharge. However, WAZ improved among children in all other cohorts. Regarding changes in linear growth, as in the larger main CHAIN cohort study, stunting was largely unchanged from admission to post-discharge day 180. Faltering in linear growth following a serious childhood illness has also been reported in other cohorts [23].

### Clinical, microbiologic, and sociodemographic factors associated with TB disease classification

Multivariate analysis was performed to identify demographic and clinical variables associated with the classification of bacteriologically confirmed and clinically diagnosed TB. When compared to children classified as unlikely TB, children classified as clinically diagnosed TB had an increased relative risk ratio for severe wasting, failure to thrive, and exhibiting a moderate illness severity score at study enrolment (**Table 5**). No variables were significantly associated with bacteriologically confirmed TB.

## DISCUSSION

Our study details the prevalence of TB among young hospitalised children in two TB-endemic countries, as well as the incidence of TB in the six months following hospital discharge. As we performed TB diagnostic evaluations of children hospitalised with any serious illness regardless of presenting signs and symptoms, our findings uniquely contrast with prior studies that sought to diagnose TB in infants and children with presumptive TB based on clinical or radiological grounds [9,24]. Overall, a high proportion (10%) of children were diagnosed and/or treated for TB, most frequently based on clinical criteria. Notably, over half (n/N = 19/37) of the children treated for TB had treatment initiated during the six-month post-discharge period and were identified using structured monitoring for the emergence of TB signs, symptoms, and new TB exposures. It is unclear if children diagnosed with TB during the post-discharge period had underlying subclinical disease that went unrecognised during their index hospitalisation or if these infections represented new acquisition or reactivation of TB in the post-discharge period. Regardless, many of these post-discharge cases of TB may have been missed in facilities that do not specifically screen for signs and symptoms of paediatric TB during follow-ups after hospital discharge.

The new availability of the Xpert Ultra assay allowed some sputum-based diagnostics to be repeated using residual cryopreserved samples among children who initially had negative diagnostic studies using the Xpert MTB/RIF assay and conventional culture. Using this method, we identified eight children with positive Xpert Ultra results who were not treated for TB, all of whom had improvement in their initial illness, survived, and had robust six-month growth outcomes. All eight of these children, as well as an additional two children treated for TB, were classified as confirmed TB based on a single ‘trace’ result on the Xpert Ultra assay. This may indicate that the highly sensitive Xpert Ultra platform detects early paucibacillary disease that may not progress to overt TB even without therapy. This observation would be consistent with reports from the pre-chemotherapy era, where young children with minimal signs and symptoms of TB were observed to spontaneously resolve their disease [25,26]. Alternatively, a positive ‘trace’ result on a single sample may reflect a false-positive result, as the specificity of the Xpert Ultra assay is lower than the Xpert MTB/RIF assay [27] when compared to culture as the gold standard. Uncertainty remains about the specificity of a single trace result for TB [28] and its inclusion in the diagnostic definition of confirmed disease for adults without HIV infection. In high-risk populations such as young children, however, a trace level of detection from a single sample is considered by WHO as confirmation of disease and should be used to guide treatment decisions given the increased risk of severe and disseminated TB in early life [29], thus prioritising sensitivity over specificity. We included the collection of one sputum sample, limiting our ability to determine the reproducibility of a positive trace result, particularly among children who did not exhibit typical signs and symptoms of TB disease and improved without treatment.

There was a high prevalence of children meeting our research definition of clinically diagnosed TB (13%), a classification that required the presence of at least three commonly reported clinical criteria of paediatric TB. Several of the signs and symptoms of clinical TB, specifically recent weight loss and reduced playfulness, are commonly observed among infants and young children with a variety of active infections and are not specific to TB [30,31]. The non-specific nature of TB presentation in young children was likely a contributing factor to the low rates of initiation of TB treatment during hospitalisation among children found to meet our research definition of clinically diagnosed TB. Specifically, only 12/46 (26%) of children classified as clinically diagnosed TB by research criteria received treatment, of whom 1/12 (8%) died compared to 4/34 (12%) children not treated for clinically diagnosed TB. Among the four children who died, all presented critically ill with severe wasting and died shortly after hospital admissions (including one death during a re-admission) while being treated for non-TB bacterial pneumonia and malnutrition. Review of hospital records revealed that TB was only clinically suspected just before their deaths. The 30 children who met criteria for clinically diagnosed TB were noted to improve following treatment for other common infections (specifically bacterial pneumonia), and hospital clinicians did not initiate TB treatment. They survived through the 6-month follow-up period, although they demonstrated significantly poorer growth as compared to children classified as unlikely to have TB.

Malnutrition and/or failure-to-thrive are considered clinical criteria for paediatric TB, and thus children who meet criteria for clinically diagnosed TB often have compromised nutritional status [6,9], as observed here. Here, children classified as clinically diagnosed TB who presented with severe wasting failed to improve their WAZ, whereas WAZ improved among severely wasted children in all other classification cohorts during the post-discharge period. Although children who met our definition for clinically diagnosed TB and went untreated may have had poor growth due to underlying TB, some children may have experienced growth failure due to another unrecognised illness or infection, socio-economic disadvantages, limited access to healthcare and/or food insecurity. Establishing the relative proportions of poor growth due to TB, underlying conditions such as HIV or environmental enteric dysfunction, and/or access to appropriate foods, will be important to effectively design and prioritise interventions aimed at improving early childhood growth. The limited catch-up growth observed among children with clinically diagnosed (regardless of TB treatment) emphasises the need to implement anthropometric monitoring and provision of nutritional support in paediatric TB treatment and screening programs [32].

Our study was limited by the exclusion of 23% of eligible children who were screened for participation due to incomplete evaluations. These 121 children were more likely to be living with HIV, had higher illness severity, were more likely to die during the study period, and were recruited from the Kampala site. It is possible that exclusion of these children introduced a sampling bias that compromises our findings; specifically, the burden of TB in the most critically ill children may have been underestimated. Our inability to complete a full diagnostic evaluation for TB within the context of a well-supported study highlights the difficult realities of performing diagnostic evaluation for TB among young, hospitalised children in typical LMIC settings where facilities to perform portable CXR and/or oxygen, and to support critically ill children, are extremely limited. In addition, our inability to repeat Xpert Ultra testing on samples yielding ‘trace’ results to confirm findings limits our capacity to interpret these results, particularly among children with no or minimal clinical signs or symptoms of TB. Our 6-month follow-up timeframe may not have been sufficient to determine long-term growth outcomes or late-onset TB. Finally, the small number of children with confirmed TB limited our ability to identify clinical differences in this population. Despite these limitations, the performance of our study across two different TB-endemic settings, the availability of detailed clinical and anthropometric data, and the six-month survival and growth outcomes are unique strengths of our study design.

## CONCLUSIONS

Our results provide several key observations regarding the burden of TB among young, hospitalised children living in endemic settings. Overall, 10% of children were treated for TB, with over half of these cases identified in the six-month post-discharge period. Public health programs in TB endemic settings need to support post-hospitalisation follow-up care for young children that includes structured screening to detect the emergence of TB signs, symptoms, and exposures. Although the use of the highly sensitive Xpert Ultra molecular assay for MTB increased the capacity to identify children with bacteriologically confirmed TB, results at the lowest level of detection must be considered in the context of the overall clinical presentation, exposure history, and pretest probability of TB. Finally, hospitalised children who meet criteria for clinically diagnosed TB are at high risk for poor growth outcomes, regardless of whether TB treatment is initiated. Healthcare systems in TB endemic settings should prioritise implementation of structured post-discharge TB screening and nutritional assessment, and support programs for all young children following hospitalisation for a serious illness.

## Additional material


Online Supplementary Document
