# Supplementary material for: Prevalence, incidence, and outcome of tuberculosis among young hospitalised children with acute illness in Sub-Saharan Africa and South East Asia
Source: J Glob Health. 2025 Dec 29;15:04338. doi: 10.7189/jogh.15.04338 (PMC12746532; doi:10.7189/jogh.15.04338)
Supplement: Online Supplementary Document [file jogh-15-04338-s001.pdf]

Supplement to: Christi MJ, Mupere E, Bin Shahid ASMS, Mukisa J, Mamun GMS, Lwanga C, Shaima SN, Atuhairwe M, Kabir MF, Aber P, Ssengooba W, Shahrin L, Banu S, Graham SM, Walson JL, Berkley JA, Ahmed T, Lancioni CL. Prevalence, incidence, and outcome of tuberculosis among young hospitalised children with acute illness in Sub-Saharan Africa and South East Asia. J Glob Health. 2025;15:04338.

Table S1. Reasons for TB study exclusions and ineligibility among CHAIN participants

**Total number screened for TB eligibility at index hospitalization**	**N=530**
**Children meeting study exclusion criteria:**	**44 (8.3%)**
Diagnosed with TB and received >7 days treatment prior to enrollment	1, 0.2% (of those screened)
Met clinical exclusion criteria	43, 8.1% (of those screened)
**Not eligible for analysis due to incomplete evaluation:**	**121 (22.8%)**
Chest X-ray not done	42, 7.9% (of those screened)
TST not done	5, 0.9% (of those screened)
Missing sputum	74, 14.0% (of those screened)
**Fulfilled the criteria for analysis (all investigations for TB sub-study completed)**	**365 (68.9%)**

Table S2. Demographic and clinical characteristics, and study outcomes, between children included in TB sub-study analysis and those excluded for incomplete evaluation

	**All (n=486)**	**Eligible for TB study (analyzed population) (n=365)**	**Excluded for incomplete evaluation (n=121)**	**p-value**
**Age < 1 year**	173 (35.6)	122 (33.4)	51 (42.2)	0.082

**Age > 1 year**	313 (64.4)	243 (66.6)	70 (57.8)	0.082
**Sex (% female)**	200 (41.2)	145 (39.7)	55 (45.5)	0.267
**Known HIV-exposure**	37 (7.6)	19 (5.2)	18 (14.9)	**0.001**
**Confirmed HIV infection (infant PCR+)**	16 (3.3)	11 (3.0)	5 (4.1)	0.560
**Nutritional Status at enrollment**				
**No wasting**	75 (15.4)	65 (17.8)	10 (8.3)	**0.011**
**Moderate wasting**	126 (25.9)	81 (22.2)	45 (37.2)	**0.001**
**Severe wasting or kwashiorkor**	285 (58.6)	219 (60.0)	66 (54.6)	0.291
**BCG vaccination**	383 (78.8)	293 (80.3)	90 (74.4)	0.169
**Cough > 14 days**	56 (11.6)	44 (12.1)	12 (9.9)	0.517
**Known TB contact or HHC with cough > 14 days**	54 (11.1)	44 (12.1)	10 (8.3)	0.247
**Treated for TB during CHAIN study**	37 (10.1)	37 (10.1)	0 (0.0)	1.000
**Readmission**	78 (16.1)	66 (18.1)	12 (9.9)	**0.034**
**Died during index hospitalization**	26 (5.4)	15 (4.1)	11 (9.1)	**0.035**
**Died during post-discharge period (at home or in hospital)**	15 (3.1)	9 (2.5)	6 (5.0)	0.221
**Illness Severity Score**				
**Acute 30 day score**				
**Low**	225 (46.3)	223 (61.1)	2 (1.6)	**<0.001**
**Medium**	153 (31.5)	86 (23.6)	67 (55.4)	**<0.001**
**High**	108 (22.2)	56 (15.3)	52 (43.0)	**<0.001**

Table S3. Growth changes over time

	Unlikely			Bacteriologically confirmed			Clinically diagnosed		
**Characteristic**	**Not SAM**	**SAM at D1**	**p-value**	**Not SAM**	**SAM at D1**	**p-value**	**Not SAM**	**SAM at D1**	**p-value**
Mean (SD)	(n = 127)	(n = 175)		(n = 9)	(n = 8)		(n = 8)	(n = 38)	
**Admission to Follow-up**									
Admission Weight	7.02 (1.55)	5.56 (1.65)	<0.001	6.85 (1.04)	5.24 (1.12)	0.008	6.29 (1.32)	5.19 (1.42)	0.059
Discharge weight	7.09 (1.48)	5.73 (1.53)	<0.001	6.83 (1.09)	5.66 (1.06)	0.041	6.39 (1.32)	5.59 (1.25)	0.15
Weight at Day 45	7.79 (1.55)	6.52 (1.62)	<0.001	7.27 (0.98)	5.98 (1.04)	0.030	7.14 (1.14)	6.18 (1.49)	0.085
Weight at Day 90	8.20 (1.52)	7.05 (1.61)	<0.001	7.56 (1.09)	6.56 (1.10)	0.10	7.47 (1.05)	6.48 (1.33)	0.055
Weight at Day 180	8.89 (1.59)	7.93 (1.62)	<0.001	8.54 (0.95)	7.58 (0.97)	0.066	7.77 (1.28)	7.25 (1.39)	0.3
Admission Height/Length	68 (7)	65 (7)	<0.001	68.6 (5.2)	64.4 (5.5)	0.13	66 (7)	64 (6)	0.4
Discharge Height/Length	68 (7)	65 (7)	<0.001	68.6 (5.2)	64.7 (5.6)	0.2	66.4 (6.6)	65.0 (6.3)	0.6
Height/Length at Day 45	70 (6)	67 (6)	<0.001	69.5 (4.2)	65.0 (5.0)	0.088	69.3 (5.6)	66.2 (5.9)	0.2
Height/Length at Day 90	72 (6)	69 (6)	<0.001	71.0 (4.3)	67.1 (4.5)	0.11	70.5 (5.0)	67.8 (5.6)	0.2
Height/Length at Day 180	75.1 (5.7)	72.0 (5.3)	<0.001	74.0 (4.0)	70.4 (4.2)	0.10	72.4 (5.0)	70.4 (5.7)	0.4
Admission MUAC	12.88 (1.08)	11.06 (1.31)	<0.001	12.72 (1.07)	10.56 (0.63)	<0.001	12.06 (0.75)	10.17 (1.26)	<0.001
Discharge MUAC	12.88 (1.06)	11.16 (1.15)	<0.001	12.75 (1.02)	10.79 (0.74)	<0.001	12.09 (0.64)	10.70 (1.02)	<0.001
MUAC at Day 45	13.45 (1.09)	12.12 (1.29)	<0.001	13.09 (1.14)	11.76 (0.78)	0.020	12.47 (0.57)	11.52 (1.29)	0.006
MUAC at Day 90	13.76 (1.05)	12.58 (1.29)	<0.001	13.38 (1.31)	12.24 (1.02)	0.083	12.54 (0.79)	11.89 (1.14)	0.10
MUAC at Day 180	14.01 (1.14)	13.12 (1.24)	<0.001	14.13 (1.41)	12.85 (0.52)	0.041	13.03 (1.05)	12.37 (1.26)	0.2
Admission WAZ	-1.99 (1.29)	-3.75 (1.26)	<0.001	-2.15 (1.03)	-4.36 (0.82)	<0.001	-3.23 (1.18)	-4.37 (1.27)	0.033
Discharge WAZ	-1.96 (1.25)	-3.62 (1.14)	<0.001	-2.22 (1.12)	-3.92 (0.74)	0.002	-3.15 (1.09)	-3.97 (1.14)	0.086
Day 45 WAZ	-1.63 (1.23)	-3.07 (1.35)	<0.001	-2.02 (1.33)	-3.57 (0.98)	0.023	-2.77 (1.18)	-3.65 (1.38)	0.12
Day 90 WAZ	-1.51 (1.21)	-2.78 (1.37)	<0.001	-2.03 (1.36)	-3.21 (1.12)	0.088	-2.67 (1.03)	-3.53 (1.28)	0.086
Day 180 WAZ	-1.41 (1.23)	-2.37 (1.38)	<0.001	-1.60 (1.09)	-2.83 (0.85)	0.026	-2.74 (0.94)	-3.18 (1.31)	0.3

Table S3. Growth changes over time

	Unlikely			Bacteriologically confirmed			Clinically diagnosed		
**Characteristic**	**Not SAM**	**SAM at D1**	**p-value**	**Not SAM**	**SAM at D1**	**p-value**	**Not SAM**	**SAM at D1**	**p-value**
Mean (SD)	(n = 127)	(n = 175)		(n = 9)	(n = 8)		(n = 8)	(n = 38)	
Admission HAZ/LAZ	-1.56 (1.44)	-2.96 (1.44)	<0.001	-1.62 (1.42)	-3.26 (1.28)	0.024	-3.04 (1.14)	-3.33 (1.72)	0.6
Discharge HAZ/LAZ	-1.65 (1.45)	-3.04 (1.43)	<0.001	-1.70 (1.39)	-3.38 (1.27)	0.020	-3.13 (1.16)	-3.27 (1.56)	0.8
Day 45 HAZ/LAZ	-1.81 (1.33)	-3.10 (1.35)	<0.001	-1.88 (1.55)	-3.41 (1.28)	0.057	-3.07 (1.28)	-3.54 (1.42)	0.4
Day 90 HAZ/LAZ	-1.79 (1.33)	-2.99 (1.32)	<0.001	-1.97 (1.54)	-3.30 (1.27)	0.090	-3.14 (1.41)	-3.46 (1.33)	0.6
Day 180 HAZ/LAZ	-1.77 (1.24)	-2.78 (1.33)	<0.001	-2.06 (1.54)	-3.54 (1.37)	0.063	-3.08 (1.10)	-3.56 (1.48)	0.3
Admission WHZ/WLZ	-1.45 (1.15)	-2.76 (1.34)	<0.001	-1.68 (0.94)	-3.60 (0.64)	<0.001	-2.08 (1.15)	-3.53 (1.33)	0.009
Discharge WHZ/WLZ	-1.34 (1.12)	-2.49 (1.19)	<0.001	-1.72 (1.02)	-2.76 (0.81)	0.035	-1.85 (0.95)	-2.94 (1.00)	0.015
Day 45 WHZ/WLZ	-0.84 (1.08)	-1.78 (1.43)	<0.001	-1.38 (1.04)	-2.22 (1.18)	0.2	-1.52 (1.00)	-2.33 (1.43)	0.10
Day 90 WHZ/WLZ	-0.75 (1.06)	-1.57 (1.42)	<0.001	-1.39 (0.95)	-1.96 (1.31)	0.4	-1.39 (0.68)	-2.33 (1.30)	0.015
Day 180 WHZ/WLZ	-0.72 (1.13)	-1.29 (1.48)	<0.001	-0.79 (1.32)	-1.32 (0.77)	0.3	-1.55 (0.90)	-1.83 (1.14)	0.5
**Post-discharge**									
**Rate of weight gain (gm/kg/day)**									
Weight gain : day1 to 45	1.95 (1.46)	3.30 (3.07)	<0.001	1.50 (1.63)	3.28 (2.40)	0.13	2.03 (1.45)	2.90 (2.48)	0.2
Weight gain : day45 to 90	1.26 (1.31)	1.84 (2.37)	0.012	0.72 (0.88)	1.98 (1.67)	0.11	1.11 (0.70)	1.71 (2.12)	0.2
Weight gain : day90 to 180	0.91 (0.54)	1.38 (1.16)	<0.001	1.51 (2.11)	1.70 (0.88)	0.8	0.64 (0.65)	1.32 (1.31)	0.064
Weight gain : day1 to 90	1.71 (1.08)	2.89 (2.52)	<0.001	1.32 (1.26)	2.88 (1.91)	0.10	1.67 (1.13)	2.65 (2.40)	0.12
Weight gain : day1 to 45	1.40 (0.77)	2.44 (1.83)	<0.001	1.49 (1.00)	2.46 (1.31)	0.12	1.38 (0.62)	2.24 (1.84)	0.044
**Rate of length gain (mm/day)**									
Length gain : day1 to 45	0.29 (0.20)	0.33 (0.29)	0.2	0.25 (0.26)	0.26 (0.17)	>0.9	0.35 (0.25)	0.25 (0.21)	0.3
Length gain : day45 to 90	0.38 (0.22)	0.41 (0.28)	0.3	0.37 (0.19)	0.42 (0.19)	0.6	0.28 (0.23)	0.42 (0.29)	0.2
Length gain : day90 to 180	0.34 (0.13)	0.37 (0.19)	0.10	0.32 (0.14)	0.33 (0.13)	>0.9	0.29 (0.11)	0.28 (0.17)	0.8
Length gain : day1 to 90	0.32 (0.16)	0.36 (0.24)	0.13	0.30 (0.20)	0.33 (0.14)	0.8	0.32 (0.22)	0.33 (0.20)	0.9
Length gain : day1 to 45	0.33 (0.11)	0.37 (0.19)	0.050	0.31 (0.15)	0.30 (0.14)	>0.9	0.32 (0.14)	0.31 (0.14)	>0.9

Table S3. Growth changes over time

Characteristic	Unlikely			Bacteriologically confirmed			Clinically diagnosed		
	Not SAM	SAM at D1	p-value	Not SAM	SAM at D1	p-value	Not SAM	SAM at D1	p-value
Mean (SD)	(n = 127)	(n = 175)		(n = 9)	(n = 8)		(n = 8)	(n = 38)	
**Anthropometric changes**									
MUAC change: day1 to 45	0.55 (0.58)	1.01 (1.09)	<0.001	0.45 (0.64)	1.12 (0.74)	0.087	0.49 (0.58)	1.15 (0.91)	0.029
MUAC change: day1 to 90	0.31 (0.47)	0.45 (0.67)	0.059	0.28 (0.37)	0.48 (0.81)	0.6	0.06 (0.58)	0.40 (0.61)	0.2
MUAC change: day1 to 180	0.24 (0.51)	0.50 (0.75)	<0.001	0.75 (1.56)	0.60 (0.55)	0.8	0.40 (0.69)	0.45 (1.03)	0.9
WAZ change: day1 to 45	0.33 (0.54)	0.65 (0.86)	<0.001	0.15 (0.44)	0.71 (0.61)	0.068	0.41 (0.43)	0.57 (0.77)	0.5
WAZ change: day1 to 90	0.12 (0.41)	0.27 (0.60)	0.015	-0.01 (0.33)	0.36 (0.52)	0.14	0.10 (0.21)	0.23 (0.57)	0.3
WAZ change: day1 to 180	0.08 (0.36)	0.34 (0.67)	<0.001	0.42 (1.26)	0.50 (0.43)	0.9	-0.04 (0.48)	0.30 (0.77)	0.2
HAZ change: day1 to 45	-0.29 (0.42)	-0.18 (0.58)	0.092	-0.32 (0.33)	-0.39 (0.33)	0.7	0.07 (0.35)	-0.35 (0.53)	0.024
HAZ change: day1 to 90	0.02 (0.34)	0.09 (0.45)	0.11	-0.09 (0.28)	0.11 (0.30)	0.2	-0.07 (0.28)	0.15 (0.51)	0.14
HAZ change: day1 to 180	0.00 (0.43)	0.18 (0.59)	0.004	-0.09 (0.50)	-0.01 (0.31)	0.7	0.01 (0.48)	-0.11 (0.65)	0.6
WHZ change: day1 to 45	0.61 (0.78)	0.98 (1.16)	0.002	0.34 (0.51)	1.35 (0.92)	0.030	0.44 (0.55)	1.09 (1.36)	0.052
WHZ change: day1 to 90	0.09 (0.56)	0.20 (0.89)	0.2	-0.01 (0.63)	0.26 (0.63)	0.4	0.13 (0.39)	0.09 (0.83)	0.8
WHZ change: day1 to 180	0.04 (0.52)	0.20 (0.83)	0.049	0.60 (1.60)	0.63 (0.71)	>0.9	-0.09 (0.76)	0.43 (1.08)	0.2

Table S4: Timing of TB Clinical Diagnosis and Initiation of Treatment

Diagnostic setting	Confirmed TB* (n=9)	Unconfirmed TB** (n=12)	Unlikely TB⅄ (n=16)
Index CHAIN hospital admission	5	5	8

Re-admission¶ to hospital	1	2	
Scheduled CHAIN D45, D90, or D180 visit	3	5	8
Unscheduled sick visit	0	0	

*Among children with confirmed TB, 9 of 17 received TB treatment

**Among children with unconfirmed TB, 12 of 46 received TB treatment

⅄Among children with unlikely TB, 16 of 302 received TB treatment

¶ Excludes children admitted from outpatient clinic specifically for TB evaluation

Table S5: Sputum Xpert MTB/RIF and Xpert Ultra results

**Result**	**Xpert MTB/RIF N=180***	**Xpert Ultra N=219***
**MTB detected**	4	13
**High**	-	2
**Medium**	-	0
**Low**	-	1
**Very low**	-	0
**Trace**	-	10
**MTB not detected**	175	206
**MTB indeterminate**	0	0
**Rifampicin resistance**	-	-
**Detected**	0	0
**Not detected**	4	2
**Indeterminate**	1	11

***34 children had their sputum sample tested using both the Xpert MTB/RIF and Xpert Ultra platforms**

Table S6. Clinical summary of children with positive sputum testing for MTB

**Site**	**Culture/ Xpert**	**Timing of test in relation to initial presentation**	**CXR findings**	**Real-time or delayed sputum results**	**TB treatment decision**

BD	Ultra	Admission	Abnormal	Delayed	Treated: presence of TB-related signs and symptoms during initial hospitalization.
BD	Ultra	Admission	Abnormal	Delayed	Treated: presence of TB-related signs and symptoms during initial hospitalization.
BD	Ultra	Re-admission	Abnormal	Delayed	Not treated: Infant lethargic on presentation but improved with treatment for severe dehydration. No other TB-related clinical symptoms.<br>Well nourished at D180.
BD	MTB/RIF	Day 45	Abnormal	Delayed	Treated: TB-related signs and symptoms during follow-up.
BD	Ultra	Admission	Abnormal	Real-time	Treated: presence of TB-related signs and symptoms during initial hospitalization.
BD	Ultra	Admission	Abnormal	Delayed	Not treated: no clinical features of TB on admission. Mild infiltrates on CXR. Well nourished at D180.
BD	Ultra	Admission	Normal	Delayed	Not treated: no clinical features of TB on admission.. Well nourished at D180.
BD	Ultra	Admission	Normal	Delayed	Not treated: no clinical features of TB on admission.. Well nourished at D180.
BD	Ultra	Admission	Normal	Delayed	Not treated: no clinical features of TB on admission. Well nourished at D180.
BD	Ultra	Admission	Abnormal	Delayed	Not treated: prolonged cough only on admission with mild infiltrates on CXR. Well nourished at D180.
BD	Culture and Xpert	Day 45	Abnormal	Delayed	Treated: developed TB-related signs and symptoms during follow-up.
BD	Ultra	Admission	Normal	Delayed	Not treated: no TB-related clinical features on admission.<br>Well nourished at D180.
UG	Ultra	Admission	Abnormal	Delayed	Not treated: no TB-related clinical features on admission.<br>Well nourished at D180.
UG	MTB/RIF	Admission	Abnormal	Real-time	Treated: presence of TB-related signs and symptoms during initial hospitalization.
UG	Ultra	Day 45	Abnormal	Real-time	Treated: developed TB-related signs and symptoms during follow-up.
UG	MTB/RIF	Admission	Abnormal	Real-time	Treated: TB-related signs and symptoms during initial hospitalization.
UG	MTB/RIF	Admission	Abnormal	Real-time	Treated: presence of TB-related signs and symptoms at initial hospitalization.

BD; Bangladesh; UG: Uganda

Annex S1

CHAIN Radiology Results CRF V1.59

CHAIN Number [5][0] [0][0][1] [ ][ ][ ]

RADIOLOGY

The Chest X ray SOP states that lateral view should only be done if antero-posterior view is NORMAL. X-rays should ideally be reviewed by 2 clinicians who are trained and / or experienced in x-ray interpretation. The original x-rays should also be stored for future review e.g. by scanning or digital copy obtained.

**CHEST X RAY 1: ALL SITES**					
**☐ Admission ☐ Deterioration ☐ Readmission ☐ Other**					
__ __/ __ __/ __ __ __ __<br>*D D / M M / Y Y Y Y*	Rotated?<br>Y N	Too Dark<br>Y N	Too light<br>Y N	**Repeated?**<br>Y N	**Lateral view?**<br>Y N
**Result after review by 2 clinicians**	☐ Normal	☐ Abnormality on left	☐ Abnormality on right	☐ Bilateral abnormality	☐ Abnormality seen on lateral view ONLY
**Abnormality, select all that apply**	☐ Air space opacification/ consolidation	☐ Air bronchogram	☐ Infiltrates	☐ Lobar collapse	☐ Tracheal displacement
	☐ Airway compression	☐ Soft tissue density suggesting lymphadenopathy	☐ Pleural effusion	☐ Nodular picture / Miliary	☐ Signs of failure or fluid overload
**Other abnormality**	☐ Cardiomegaly	☐ Rib fracture	☐ Mediastinal mass	☐ Pneumothorax	☐ Rib features consistent with rickets
	Other				
